# pH-Responsive Intra- and Inter-Molecularly Micelle Formation of Anionic Diblock Copolymer in Water

**DOI:** 10.3390/polym8020056

**Published:** 2016-02-19

**Authors:** Masanobu Mizusaki, Yoshihiko Shimada, Yotaro Morishima, Shin-ichi Yusa

**Affiliations:** 1 Department of Applied Chemistry, Graduate School of Engineering, University of Hyogo, 2167 Shosha, Himeji, Hyogo 671-2280, Japan; yusa@eng.u-hyogo.ac.jp; 2 Faculty of Engineering, Fukui University of Technology, 6-3-1 Gakuen, Fukui 910-8505, Japan; morisima@fukui-ut.ac.jp

**Keywords:** block copolymer, micelles, RAFT polymerization, water-soluble polymer, pH-responsive

## Abstract

Poly(sodium2-(acrylamido)-2-methylpropanesulfonate)-*block*-poly(sodium11-(acrylamido)undecanoate) (PAMPS–PAaU) was synthesized via reversible addition-fragmentation chain transfer (RAFT)-controlled radical polymerization. The “living” polymerization of PAaU was evidenced by the fact that the molecular weight distribution was narrow (*M*_w_/*M*_n_ = 1.23). The pH-induced association behavior of PAMPS–PAaU in 0.1 M NaCl aqueous solutions as a function of solution pH was investigated by ^1^H NMR spin-spin relaxation time, dynamic light scattering (DLS), static light scattering (SLS), and fluorescence probe techniques. These results indicated that PAMPS–PAaU formed polymer micelles in 0.1 M NaCl aqueous solutions at pH < 9. At pH = 8–9, the polymer formed the micelles intramolecularly due to hydrophobic self-association of the PAaU block within the single polymer chain. On the other hand, at pH < 8, micellization occurred intermolecularly to form polymer micelles comprising hydrophobic PAaU cores and hydrophilic PAMPS shells.

## 1. Introduction

Amphiphilic diblock copolymers form micelles in water in which hydrophobic blocks constitute a core and hydrophilic blocks form shells [1]. The amphiphilic diblock copolymer micelles are important in many applications, including separation [2] and delivery systems [3]. In recent years, external stimuli-responsive block copolymers have attracted considerable interest. Remarkable progress has been made in the development of block copolymers responding to physical and chemical stimuli such as heat, pH, light, and added electrolytes [4,5,6].

Progress in polymer synthesis techniques makes it possible to produce various water-soluble block copolymers with controlled structures. Radical polymerization methods that have been used to synthesize well-defined polymers include nitroxide-mediated or stable free radical polymerization (SFRP) [7], atom transfer radical polymerization (ATRP) [8,9], and reversible addition-fragmentation chain transfer (RAFT) methods [10]. Above them, the advantage of RAFT polymerization is applicable to a wide range of monomers under a broad range of experimental conditions. We previously synthesized poly(sodium2-(acrylamido)-2-methylpropanesulfonate)-*block*-poly(sodium6-(acrylamido)hexanoate) (PAMPS–PAaH) via RAFT radical polymerization, and investigated its pH-induced association behavior in 0.1 M NaCl aqueous solutions with dynamic light scattering (DLS), static light scattering (SLS), ^1^H NMR relaxation, and fluorescence probe techniques [11]. The experimental data indicated that PAMPS–PAaH forms polymer micelles intermolecularly in 0.1 M NaCl aqueous solutions at pH < 4 whereas the micelles were dissociated at high pH conditions. The pH-induced association and dissociation are completely reversible without hysteresis [11].

**Chart 1:**
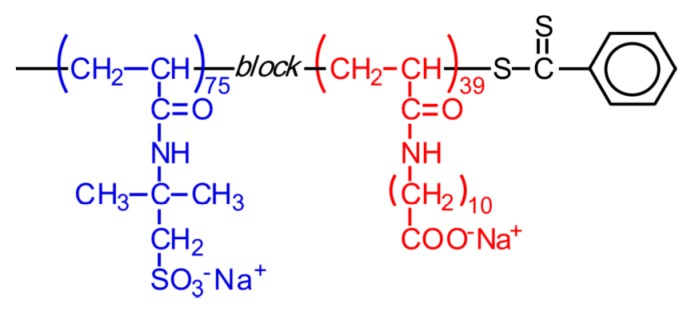
Chemical structure of diblock copolymer (PAMPS–PAaU). The most probable end group structure is indicated.

In this paper, we report on the pH-induced self-association behavior of a diblock copolymer (PAMPS–PAaU) of poly(sodium2-(acrylamido)-2-methylpropanesulfonate) (PAMPS) and poly(sodium11-(acrylamido)undecanoate) (PAaU), which has a longer pendant alkyl chain than that of PAaH, in an aqueous solution by the RAFT process using a PAMPS macro-chain transfer agent (Chart 1). The diblock copolymer will exhibit pH-induced self-association due to the selective protonation of the carboxylate residues at low pH values. We mainly focused on the pH-responsive association and dissociation behavior of PAMPS–PAaU in aqueous solution as characterized using DLS, SLS, ^1^H NMR relaxation time, and fluorescence probe techniques. We expected that the micellization due to the hydrophobic self-association of PAMPS–PAaU occurs at relatively higher pH compared with PAMPS–PAaH because the pKa value for the PAaU block is higher than that of the PAaH block.

## 2. Experimental Section

### 2.1. Reagents

The 4-cyanopentanoic acid dithiobenzoate (CPD) was synthesized according to the method reported by McCormick and co-workers [12]. The 2-(acrylamido)-2-methyl propanesulfonic acid (AMPS), acryloyl chloride (98%), and 4,4′-azobis(4-cyanopentanoic acid) (V-501) from Wako Pure Chemical (Osaka, Japan) were used as received without further purification. The 8-anilino-1-naphthalenesulfonic acid ammonium salt hydrate (ANS) and 11-aminoundecanoic acid (97%) from Sigma-Aldrich (St. Louis, MO, USA) was used as received without further purification. Methanol was dried over 4 Å molecular sieves and distilled. Water was purified with a Millipore Milli-Q system (Billerica, MA, USA). Other reagents were used as received.

### 2.2. Synthesis of Sodium 11-(Acrylamido)undecanoate (AaU)

Acryloyl chloride (56.7 g, 0.625 mol) was added to 0.6 M NaOH aqueous solution (1.5 L) of 11-aminoundecanoic acid (40.3 g, 0.20 mol) over a period of 30 min in an ice bath. The solution was stirred for 3 h at room temperature. After the reaction, the solution pH was changed to pH 3 using 6 M hydrochloric acid. The precipitate was filtered, washed twice with water. The crude product was purified by recrystallization from a mixture of acetone and *n*-hexane (1/3, *v*/*v*) three times and dried at 50 °C under vacuum, and finally 15.4 g (30.4%) of 11-(acrylamido)undecanoic acid was obtained. AaU was prepared by neutralization of 11-(acrylamido)undecanoic acid (15.4 g, 60.7 mmol) with an equivalent of NaOH (2.43 g, 60.8 mmol) in methanol followed by precipitation of the salt with diethyl ether. The sample was dried at 50 °C under vacuum, and finally 16.1 g (95.7%) of AaU was obtained.

### 2.3. Preparation of PAMPS Macro-Chain Transfer Agent (PAMPS Macro-CTA)

AMPS (25.0 g, 121 mmol) was neutralized with NaOH (4.81 g, 121 mmol) in 60 mL of water, and CPD (232 mg, 0.829 mmol) and V-501 (46.4 mg, 0.166 mmol) were added to this solution. The mixture was degassed by purging with Ar gas for 30 min. Polymerization was carried out at 70 °C for 4 h. The polymer was dialyzed against pure water for a week and recovered by a freeze-drying technique (yield 23.2 g, conversion 83.9%). The obtained PAMPS could be used as a macro-CTA.

### 2.4. Block Copolymerization

PAMPS macro-CTA (2.43 g, 0.14 mmol), AaU (2.06 g, 7.43 mmol), and V-501 (4.63 mg, 0.0165 mmol) were dissolved in 13.5 mL of water. The solution was deoxygenated by purging with Ar gas for 30 min. Block copolymerization was carried out at 70 °C for 4 h. The diblock copolymer was purified by dialysis against a NaOH aqueous solution at pH 8 for a week, changing the dilute alkaline aqueous solution twice a day. The diblock copolymer (PAMPS–PAaU) was recovered by a freeze-drying technique (yield 3.52 g, conversion 89.4%). Gel-permeation chromatography (GPC) for the reaction mixture was measured to estimate the number-average molecular weight (*M*_n_) and molecular weight distribution (*M*_w_/*M*_n_).

### 2.5. Measurements

GPC analysis was performed at 40 °C with a reflective index (RI) detector equipped with a Shodex 7.0 μm bead size GF-7F HQ column (exclusion limit ~10^7^) using a phosphate buffer solution at pH 9 containing 10 *v* % acetonitrile as an eluent at a flow rate of 0.6 mL/min. *M*_n_, weight-average molecular weight (*M*_w_), and *M*_w_/*M*_n_ of the polymer were calibrated with a standard poly(sodium styrenesulfonate) samples of 11 different molecular weights ranging from 1.37 × 10^3^ to 2.61 × 10^6^.

DLS data were obtained at 25 °C with an Otsuka Electronics Photal DLS-7000DL light scattering spectrometer (Osaka, Japan) equipped with an ALV-5000E multi-τ digital time correlator. A He–Ne laser (10.0 mW at 632.8 nm) was used as a light source. The intensity-intensity time correlation *g*^(2)^(*t*) in the self-beating mode was measured, and *g*^(2)^(*t*) is related to the normalized autocorrelation function of the scattered electric field *g*^(1)^(*t*):

(1) $$g^{\left(2\right)}(t)=B\;\left(1+\beta \;{\left|g^{\left(1\right)}(t)\right|}^{2}\right)$$

where *B* is a baseline, β is a factor which takes into account deviations from ideal correlation, and *t* is the delay time. For a polydisperse sample, *g*^(1)^(*t*) is related to the relaxation time distribution, τ*A*(τ). To obtain τ*A*(τ), the inverse Laplace transform (ILT) analysis was performed using a constrained regularization routine, REPES [13,14].

(2) g(1)(t)= ∫τA(τ) exp (−tτ) d ln τ

Here, τ is the relaxation time. The relaxation time distributions are given as a τ*A*(τ) *vs.* log(τ) profile with an equal area. The translational diffusion coefficient (*D*) is calculated from *D* = (Γ/*q*^2^)*_q_*_→0_, where Γ is the relation rate and *q* = (4π*n*/λ)sin(θ/2) with *n* being the refractive index of solvent, λ being the wavelength (632.8 nm), and θ being the scattering angle. The hydrodynamic radius (*R*_h_) is calculated using the Einstein–Stokes relation *R*_h_ = *k*_B_*T*/6πη*D*, where *k*_B_ is Boltzmann’s constant, *T* is the absolute temperature, and η is the solvent viscosity.

SLS measurements were performed at 25 °C with an Otsuka Electronics Photal DLS-7000DL light scattering spectrometer equipped with a He–Ne laser (10.0 mW, 632.8 nm). *M*_w_, *z*-average radius of gyration (*R*_g_), and the second virial coefficient (*A*_2_) values were estimated from the relation [15].

(3) $$\frac{KC_{p}}{R_{\theta }}=\frac{1}{M_{w}}\left(1+\frac{1}{3}{R_{g}}^{2}q^{2}\right)+2A_{2}C_{p}$$

where *C*_P_ is the polymer concentration, *R*_θ_ is the Rayleigh ratio, and *K* = 4π^2^*n*^2^(d*n*/d*C*_P_)2/*N*_A_λ^4^ with d*n*/d*C*_P_ being the refractive index increment against *C*_P_ and *N*_A_ being Avogadro’s number. By measuring *R*_θ_ for a set of *C*_P_ and θ, value of *M*_w_, *R*_g_, and *A*_2_ were estimated from Zimm plots. Benzene was used for the calibration of the instrument. Values of d*n*/d*C*_P_ were determined with an Otsuka Electronics Photal DRM-1020 differential refractometer (Osaka, Japan) at 25 °C.

The ^1^H NMR spectra were obtained with a Bruker DRX-500 spectrometer (Billerica, MA, USA) operating at 500 MHz in D_2_O. Chemical shifts were determined by using 3-(trimethylsilyl)propionic-2,2,3,3-*d*_4_ acid as an internal reference. The ^1^H NMR spin-spin relaxation times (*T*_2_) were determined by the Carr-purcell-Meiboom-Gill (CPMG) method [16]. A 90° pulse of 13.85 μs was calibrated and used for the measurement. Peak intensities at 12 different numbers of 180° pulse were measured.

Fluorescence spectra were recorded on a Hitachi F-2500 fluorescence spectrophotometer (Tokyo, Japan). ANS was used as a fluorescence probe. A 0.1 M NaCl aqueous stock solution of ANS (1.89 × 10^−4^ M) was prepared. Emission spectra of ANS were measured with excitation at 350 nm. Excitation and emission slit widths were maintained at 20 and 5.0 nm, respectively.

### 2.6. Preparation of Sample Solutions

The solution pH was adjusted by adding a proper amount of aqueous NaOH or HCl. All measurements were made with 0.1 M NaCl aqueous solutions to avoid an effect of a small change in the ion strength resulting from a small amount of NaOH or HCl added to the solutions. For DLS measurements, polymer solutions containing 0.1 M NaCl were employed to minimize interpolymer electrostatic interactions. The polymer concentration was 10 g/L. SLS and d*n*/d*C*_P_ measurements were performed at *C*_P_ ranging from 1.0 to 10 g/L in 0.1 M NaCl aqueous solutions. Sample solutions for DLS and SLS measurements were filtered with a 0.2 μm pore size membrane filter. The sample solutions of the diblock copolymer at *C*_P_ = 10 g/L for ^1^H NMR measurements were prepared in D_2_O containing 0.1 M NaCl, and pD was adjusted with a D_2_O solution of NaOD or DCl. The final pD value was determined from the relation pD = pH + 0.4 [17].

## 3. Results and Discussion

**Figure 1:**
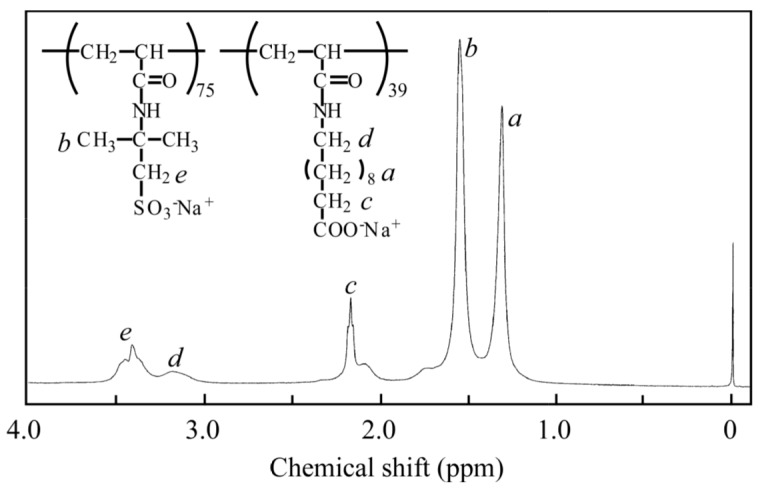
The 500 MHz ^1^H NMR spectrum for PAMPS-PAaU at *C*_p_ = 10 g/L in D_2_O containing 0.1 M NaCl at pH 10.

We prepared the diblock copolymer, PAMPS–PAaU, via a RAFT technique using PAMPS macro-CTA. The degree of polymerization (DP) of PAMPS macro-CTA, which was determined from GPC, was 75, because the terminal phenyl protons in PAMPS macro-CTA were overlapped with the pendant amide groups. The DP of the PAaU block was 39, calculated from ^1^H NMR peak area intensities of 3.4 ppm derived from a methylene proton in the PAMPS block (*e*) and an area at around 3.2 ppm derived from a methylene proton in the PAaU block (*d*), which is shown in Figure 1. *M*_n_ and *M*_w_/*M*_n_ of PAMPS–PAaU, estimated from GPC, were 7.63 × 10^4^ and 1.23, respectively. The results for characteristics of PAMPS–PAaU are listed in Table 1. The *M*_w_/*M*_n_ value of PAMPS–PAaU is relatively small (*M*_w_/*M*_n_ = 1.23), predicting that the controlled/living polymerization proceeded successfully [11].

**Figure 2:**
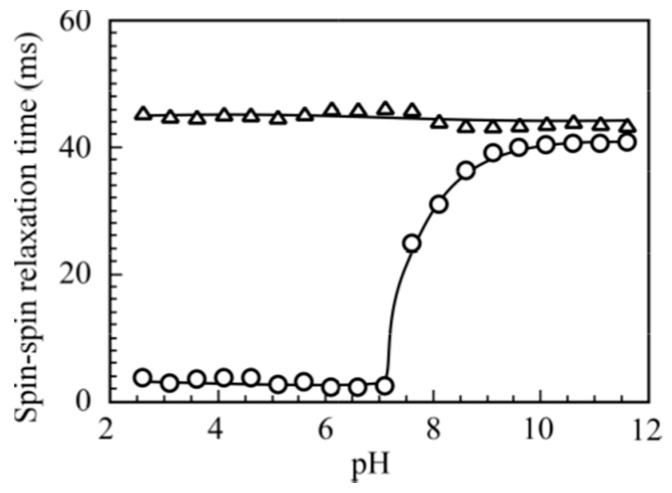
Spin-spin relaxation time (*T*_2_) for the pendant methylene protons in the PAaU block at 3.2 ppm (○) and the pendant methylene protons in the PAMPS block at 3.4 ppm (△) in PAMPS–PAaU as a function of pH in D_2_O containing 0.1 M NaCl at *C*_p_ = 10 g/L.

To examine the pH-induced self-association behavior of PAMPS–PAaU, evaluation of the ^1^H NMR relaxation time is a useful tool, because *T*_2_ decreases as the molecular motion decreases. To obtain information about motional restriction of the pendant methylene protons in the PAaU block and the PAMPS block, when the polymer forms core–shell-type micelle, we performed ^1^H NMR relaxation time measurements with the diblock copolymer in D_2_O containing 0.1 M NaCl aqueous solutions under various pH conditions. In Figure 2, *T*_2_ values for the diblock copolymer observed at around 3.2 ppm derived from the pendant methylene proton in the PAaU block and at around 3.4 ppm derived from the pendant methylene proton in the PAMPS block are plotted against solution pH. In the case of the methylene proton in the PAaU block, the *T*_2_ value is virtually constant at 40 ms at pH > 9. Upon the decrease in solution pH from 9 to 7, the *T*_2_ value is sharply decreased, reaching a constant value of 4 ms. The observation indicates that a transition from unimer to micelle, and *vice versa*, occurs within a range of pH between 7 and 9. In contrast, the *T*_2_ value of the methylene proton in the PAMPS block shows a constant value of 45 ms in the whole range of solution pH, indicating that the PAMPS block is not incorporated into the micelle core.

**Figure 3:**
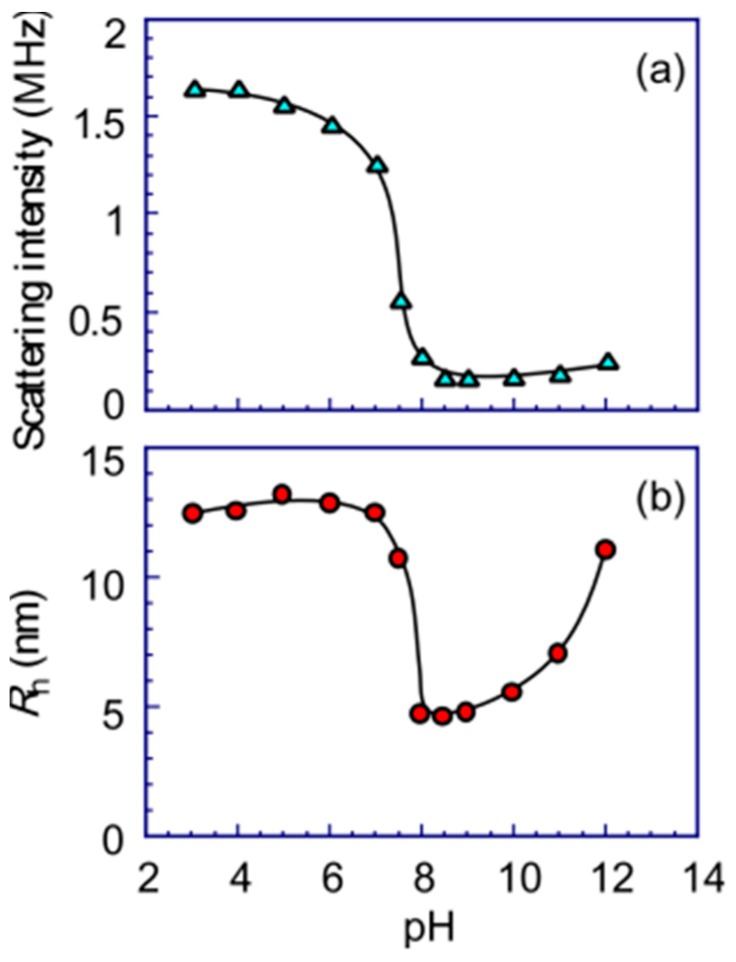
(**a**) Scattering intensity and (**b**) hydrodynamic radius (*R*_h_) for PAMPS–PAaU as a function of solution pH at *C*_p_ = 10 g/L in 0.1 M NaCl aqueous solutions.

The formation of the polymer micelles from the diblock copolymer under acidic condition was observed by DLS. Figure 3a compares a variation of scattering intensity for the solution of the diblock copolymer plotted against solution pH. The scattering intensity increased rapidly as the solution pH is decreased from 8.5 to 6, implying that micelle formation is occurred at pH < 8.5. The change in the scattering intensity fairly corresponds to that in the *T*_2_ value. Figure 3b compares *R*_h_ as a function of solution pH. The tendency shown in Figure 3b is quite different from that shown in Figure 3a. Above pH 8, the *R*_h_ value increases with increasing the solution pH from 8 to 12. One can estimate that an ionization of the carboxylate group in the PAaU block promotes the electrostatic repulsive force within one polymer chain at high pH, inducing the increment of the *R*_h_ value with increasing solution pH. At pH < 8, the *R*_h_ value also increases with decreasing the solution pH from 8 to 6.5. In this pH range, the number of carboxylate ions in the PAaU block decreases, inducing the micelle formation due to hydrophobic self-association of the pendant undecanoic acid groups in the PAaU block. The intermediate region around pH 8–9 is where it suggests that the reduction in ionic repulsion on the carboxylic acid containing the PAaU block results in less chain expansion in solution and/or formation of unimolecular micelles. The result in Figure 3b will be discussed below. The previous study for the diblock copolymers of PAMPS–PAaH shows that the pH range of a sharp increase in the *R*_h_ is from 5 to 4, which is lower than that for PAMPS–PAaU. The result suggests that the micelle formation occurs in the higher pH range of the solutions for PAMPS–PAaU because the pKa value for the PAaU block is higher than that of the PAaH block. The pKa values of lauric acid and hexanoic acid are 5.3 and 4.88, respectively. Fatty acid with a long alkyl chain is apt to have a high pKa value.

**Figure 4:**
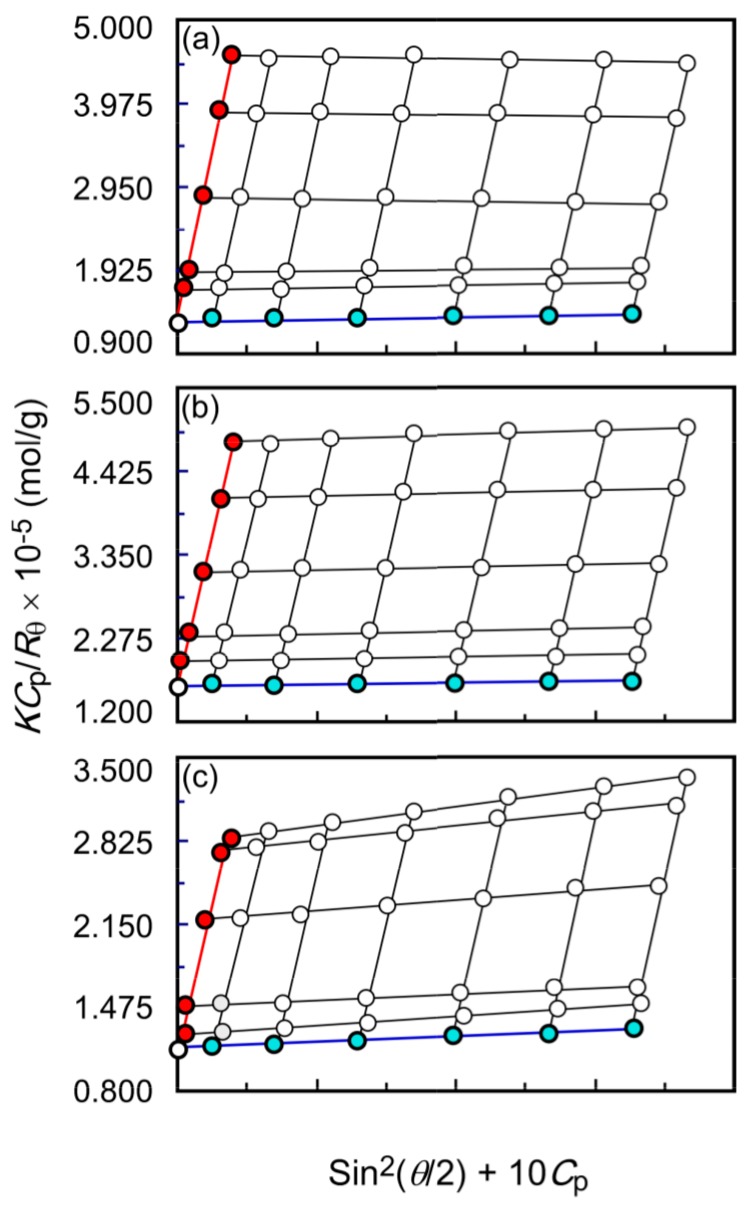
Zimm plots for PAMPS–PAaU at pH 3 (**a**), 9 (**b**), and 12 (**c**) in 0.1 M NaCl aqueous solutions. The Rayleigh ratio (*R*_θ_) was determined by subtracting the solvent scattering from the total scattering of the solution. The red and blue lines indicate the concentration (*C*_p_) dependence at scattering angle (θ) = 0 and θ dependence at scattering angle (θ) = 0, respectively.

Zimm plots for PAMPS–PAaU obtained by SLS measurements in 0.1 M NaCl aqueous solutions at pH 3, 9 and 12 in the *C*_p_ range of 1.0–10 g/L are shown in Figure 4. The SLS data for the PAMPS–PAaU polymers at pH 3, 9 and 12 are presented along with DLS data in Table 2. Values of *M*_w_ were estimated by extrapolation of *C*_p_ and θ to zero, and values of *R*_g_ and *A*_2_ were estimated from the slope of the angular and concentration dependence in the Zimm plots, respectively. A value of *N*_agg_ was calculated from the ratio of *M*_w_ values for the micelle and its unimer. *M*_w_ for the unimer was determined by GPC using a mixed solvent of water and acetonitrile (90/10, *v*/*v*) containing phosphate buffer at pH 9 as an eluent (Table 1). The *M*_w_ values determined by SLS at pH 12 and 9 fairly coincide with the *M*_w_ value determined by GPC. There is a possibility that the smaller *M*_w_ at pH 9 implies a formation of collapsed structure due to hydrophobic self-association of the PAaU block in one polymer chain. This is indicative that PAMPS–PAaU shows the unimer state in 0.1 M NaCl aqueous solutions at pH 9 and 12. On the other hand, the *M*_w_ value at pH 3 shows one order of magnitude larger than those at pH 9 and 12, anticipating that the hydrophobic self-association of the polymer chains occurs intermolecularly. The *R*_h_ as a function of solution pH, which is shown in Figure 3b, supports the results of the SLS data. The *N*_agg_ value at pH 3 was estimated to be 9.2, as presented in Table 2. The smaller *A*_2_ value is the sign of low solubility [11]. The smallest *A*_2_ value is obtained at pH 3 among pH 3, 9 and 12, indicating that the protonation of the carboxylic acid group reduces the solubility of the polymer. The *R*_g_ value is practically independent of the solution pH. The *R*_g_/*R*_h_ ratio is a parameter that depends on the polymer chain conformation and polydispersity. The theoretical value of the *R*_g_/*R*_h_ ratio for a homogeneous sphere is 0.778, and it increases substantially for a less dense structure and polydisperse solution [18]. The *R*_g_/*R*_h_ ratio for PAMPS–PAaU at pH 3, 9, and 12 are 1.20, 2.35, and 1.59, respectively. The observation suggests that the protonated PAaU block core of PAMPS–PAaU at pH 3 is the densest among pH 3, 9 and 12 in 0.1 M NaCl aqueous solutions. The *R*_g_/*R*_h_ ratios at pH 9 and 12 were larger than that at pH 3, suggesting that PAMPS–PAaU chains at pH 9 and 12 were relatively expanded chain conformations because of the electrostatic repulsions of the pendant sulfonate and carboxylate anions.

**Figure 5:**
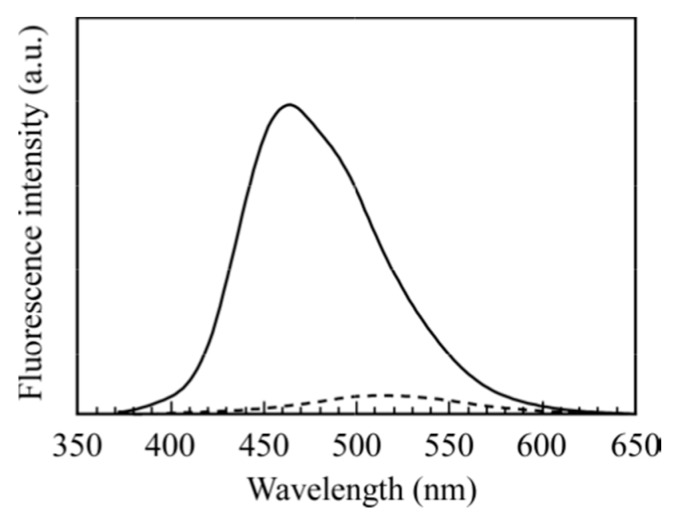
Fluorescence spectra for ANS in the absence (---) and presence (—) of PAMPS–PAaU in 0.1 M NaCl aqueous solutions at pH 3.

**Figure 6:**
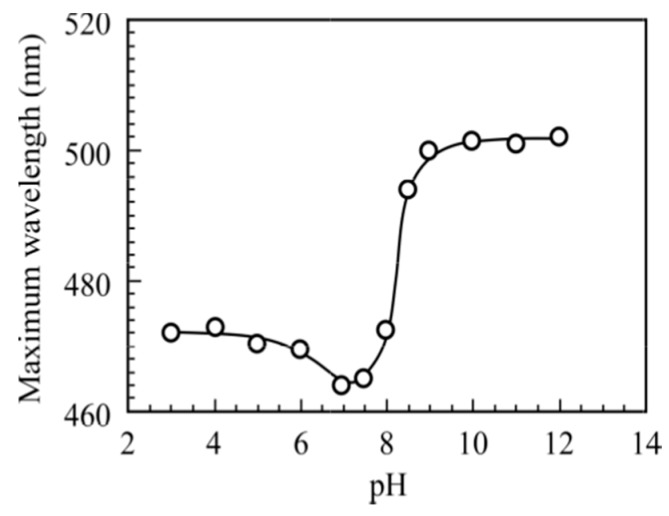
Wavelength of emission maximum in ANS fluorescence spectra as a function of solution pH in the presence of PAMPS–PAaU in 0.1 M NaCl aqueous solutions.

The fluorescence spectra of the ANS probe indicate the microscopic polarity around the probe [19]. ANS emits fluorescence strongly in nonpolar media accompanying a significant blue shift while it emits weakly in polar media. Therefore, an increased fluorescence intensity along with a blue shift of the emission maximum indicates that the probe is located in less polar media. It is also known that the fluorescence intensity and emission maximum are not influenced by changes in solution pH [11]. The fluorescence spectra for 0.2 mM of ANS in the absence and presence of PAMPS–PAaU are depicted in Figure 5 in 0.1 M NaCl aqueous solutions at pH 3. The fluorescence intensity and maximum wavelength for ANS in the presence of the diblock copolymer is remarkably increased and blue-shifted, respectively. The results suggest that the diblock copolymer micelle is able to incorporate the ANS molecules into hydrophobic microdomains formed from the self-association of the PAaU blocks. In Figure 6, the emission maximum wavelength for ANS solubilized in 0.1 M NaCl aqueous solutions in the presence of 0.35 g/L PAMPS–PAaU is plotted against the solution pH. In a high pH region (pH > 9), the emission maximum is practically constant at about 500 nm, which is a similar value for ANS in water. At pH < 9, however, the emission maximum blue shifts to 465 nm with decreasing solution pH within a pH range of 8–9, although PAMPS–PAaU shows a unimer state at this pH range according to the results of DLS and SLS. A pH value for the onset of the blue shift is higher for the PAMPS–PAaU micelle than for the diblock copolymer micelles of PAMPS–PAaH [11]. The result indicates that the micelle formation due to the hydrophobic self-association of PAMPS–PAaU occurs at higher pH than PAMPS–PAaH. In the pH range 8–9, PAMPS–PAaU is estimated to form the micelle intramolecularly. At pH < 8, PAMPS–PAaU forms an interpolymer micelle in 0.1 M NaCl aqueous solution. When the solution pH was decreased from 12 to 3 and subsequently increased back to 12, a pH-induced fluorescence spectral change was found to be completely reversible without hysteresis.

**Figure 7:**
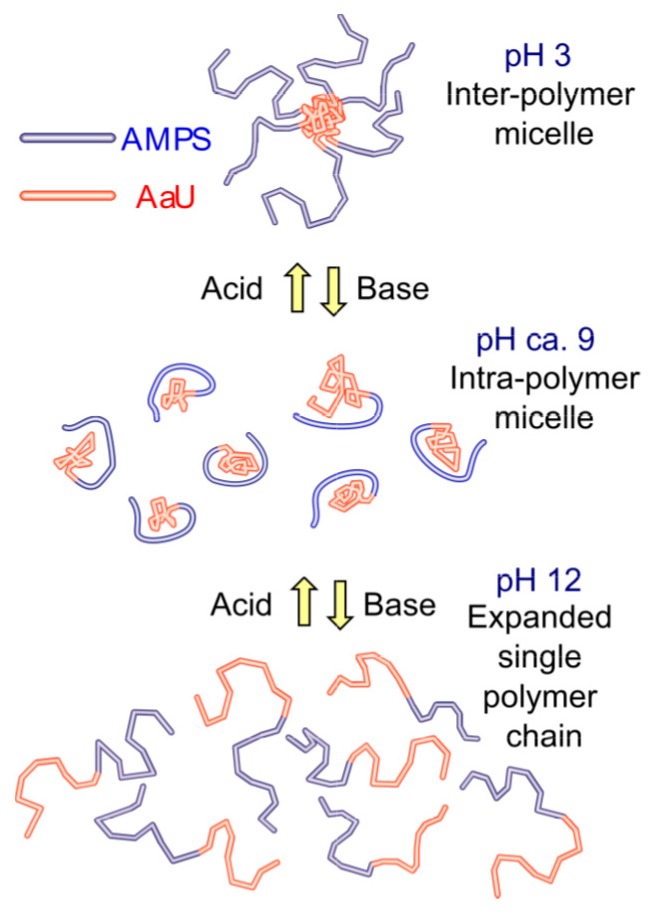
Conceptual illustration of pH-induced association behavior of PAMPS–PAaU in 0.1 M NaCl aqueous solutions.

In this study, the results for ^1^H NMR relaxation time, DLS, SLS, and fluorescence suggest that the pH-induced micelle formation and dissociation occurred for the diblock copolymer PAMPS–PAaU in 0.1 M NaCl aqueous solution. The data gives the estimation that the micelle formation occurs intramolecularly at pH = 8–9, whereas the intermolecular micelle is formed at pH < 8. We presume that the core–shell-type micelle is formed at pH < 8 [20]. The schematic illustrations of the micelle formation for PAMPS–PAaU are depicted in Figure 7 at varying pH values.

## 4. Conclusions

The diblock copolymer PAMPS–PAaU was synthesized via RAFT polymerization. The polymerization of AaU used by AMPS macro-CTA proceeded in accordance with a controlled/living mechanism by the fact of the narrow molecular weight distribution (*M*_w_/*M*_n_ = 1.23). The pH-induced association behavior of PAMPS–PAaU in 0.1 M NaCl aqueous solution at a pH range from 2 to 12 was investigated by ^1^H NMR relaxation, DLS, SLS, and fluorescence probe techniques. These experimental data indicated that PAMPS–PAaU formed polymer micelles in 0.1 M NaCl aqueous solutions at pH < 9. At pH = 8–9, the diblock copolymer formed the micelles intramolecularly due to the hydrophobic self-association of the PAaU block in one polymer chain. In contrast, the core-shell-type micelle is formed due to interpolymer hydrophobic self-association at pH < 8.

## Figures and Tables

**Table 1:** Degrees of polymerization (DP) of PAMPS and PAaU blocks, number and weight-average molecular weight (*M*_n_ and *M*_w_), and molecular weight distribution (*M*_w_/*M*_n_) of the diblock copolymer.

Sample	DP of PAMPS *^a^*	DP of PAaU *^b^*	*M*_n_ *^a^* × 10^−4^	*M*_w_ *^a^* × 10^−4^	*M*_w_/*M*_n_ *^a^*
PAMPS–PAaU	75	39	7.63	9.38	1.23

^a^ Degree of polymerization (DP) was determined by GPC eluted with a mixed solvent of water and acetonitrile (80/20, *v*/*v*) containing 0.1 M NaNO_3_; ^b^ Estimated by ^1^H NMR.

**Table 2:** Static and dynamic light scattering data for the polymer micelles formed from PAMPS–PAaU at pH 3, 9, and 12.

pH	*M*_w_^a^ × 10^−4^	*A*_2_^a^ × 10^4^m (mol·mL·g^−2^)	*R*_g_^a^ (nm)	*R*_h_^b^ (nm)	*R*_g_/*R*_h_	*N*_agg_^c^
3	78.2	1.65	15.0	12.5	1.20	9.2
9	6.1	15.6	12.0	5.1	2.35	1
12	8.5	8.86	17.8	11.2	1.59	1

^a^ Determined by SLS in 0.1 M NaCl aqueous solutions; ^b^ Determined by DLS in 0.1 M NaCl aqueous solutions; ^c^ Apparent aggregation numbers of the diblock copolymer micelles calculated from *M*_w_ of the micelles determined by SLS in 0.1 M NaCl aqueous solutions at pH 3, 9, and 12 and *M*_w_ of the corresponding unimer determined by GPC eluted with a mixed solvent of water and acetonitrile (80/20, *v*/*v*) containing in 0.1 M NaNO_3_.

## Abbreviations

SFRP: Stable free radical polymerization
ATRP: Atom transfer radical polymerization
RAFT: Reversible addition-fragmentation chain transfer
PAMPS–PAaH: Poly(sodium2-(acrylamido)-2-methylpropanesulfonate)-*block*-poly(sodium6-(acrylamide)-hexanoate)
DLS: Dynamic light scattering
SLS: Static light scattering
^1^H NMR: Proton neuclear magnetic resonance
PAMPS: Poly(sodium2-(acrylamido)-2-methylpropanesulfonate)
PAaU: Poly(sodium11-(acrylamide)hexanoate)
CPD: 4-Cyanopentanoic acid dithiobenzoate
AMPS: 2-(Acrylamido)-2-methylpropanesulfonic acid
V-501: 4,4′-Azobis(4-cyanopentanoic acid)
ANS: 8-Anilino-1-naphthalenesulfonic acid ammonium salt hydrate
AaU: Sodium11-acrylamidohexanoate
PAMPS macro-CTA: Poly(sodium 2-(acrylamido)-2-methylpropanesulfonate) macro-chain transfer agent
GPC: Gel-permeation chromatography
*M*_n_: Number-average molecular weight
*M*_w_/*M*_n_: Molecular weight distribution
RI: Refractiv index
*M*_w_: Weight-average molecular weight
B: Baseline
β: A factor which takes into account deviations from ideal correlation
*t*: Delay time
τ: Relaxation time
*D*: Translational diffusion coefficient
*q*: Scattering vectr
*n*: Refractive index
λ: Wavelength
θ: Scattering angle
*R*_h_: Hydrodynamic radius
*k*_B_: Boltzmann’s constant
*T*: Absolute temperature
η: Solvent viscosity
*R*_g_: *z*-Average radius of gyration
*A*_2_: The second virial coefficient
*C*_p_: Polymer concentration
*R*_θ_: Rayleigh ratio
*K*: Optical constant
d*n*/d*C*_p_: Refractive index increment against the polymer concentration
*N*_A_: Avogadro’s number
*T*_2_: Spin-spin relaxation time
DP: Degree of polymerization
*N*_agg_: Aggregation number
